# Dosage-dependent regulation of embryonic diapause by sorbitol dehydrogenase in the silkworm, *Bombyx mori*

**DOI:** 10.1371/journal.pgen.1011933

**Published:** 2025-10-30

**Authors:** Dongbin Chen, Dehong Yang, Xin Fu, Haixu Bian, Yongping Huang, Yanqun Liu, Zulian Liu

**Affiliations:** 1 School of Environmental Science and Engineering, Shanghai Jiao Tong University, Shanghai, China; 2 Department of Sericulture, College of Bioscience and Biotechnology, Shenyang Agricultural University, Shenyang, China; University of Kentucky, UNITED STATES OF AMERICA

## Abstract

Insects have evolved diapause to cope with harsh environmental conditions, during which their metabolism undergoes significant remodeling. The silkworm, *Bombyx mori*, enters diapause in the early embryonic stage, a process critically regulated by the sorbitol metabolism pathway and its key enzyme, sorbitol dehydrogenase (SDH). Nevertheless, the precise involvement of SDH in the diapause regulation of the silkworm remains to be fully understood. In this study, we identified that *BmSdh2* is highly expressed in diapause-destined silkworm embryos through RNA-seq analysis. Genetic manipulation of *BmSdh2* expression significantly influenced diapause progression: complete homozygous knockout of *BmSdh2* led to diapause termination, whereas partial loss-of-function mutations maintained the wild-type diapause phenotype. Furthermore, integrative LC-MS/MS, metabolomic, and lipidomic analyses demonstrated that *BmSdh2* dosage critically modulates diapause maintenance. These findings highlight *BmSdh2* as a novel and potentially central molecular regulator in the silkworm diapause pathway.

## Author summary

Diapause serves as a crucial survival mechanism in insects, enabling them to withstand extreme environmental conditions by temporarily suspending development. In the silkworm (*Bombyx mori*), diapause involves metabolic reprogramming, including the conversion of glycogen into cryoprotectants such as sorbitol. Although sorbitol dehydrogenase (SDH) is known to reverse this process during diapause termination, its precise regulatory role remains unclear. Here, we identified *BmSdh2* as a key gene that is highly expressed in diapause-destined silkworm embryos. Using CRISPR/Cas9-mediated mutagenesis, we discovered that *BmSdh2* regulates diapause in a dosage-dependent manner: a complete knockout (*BmSdh2*^*−/−*^) eliminated diapause, whereas heterozygous mutants (*BmSdh2*^*+/−*^) retained the wild-type diapause phenotype. Metabolomic and lipidomic analyses revealed that *BmSdh2* dosage critically modulates sorbitol accumulation and lipid metabolism, linking its activity to diapause maintenance. Our findings establish *BmSdh2* as a molecular switch within the silkworm diapause pathway, shedding light on how metabolic sensing governs developmental plasticity. This work not only advances our understanding of insect diapause but also holds potential for manipulating diapause in sericulture, with implications for agricultural and biotechnological applications.

## Introduction

Organisms in temperate and polar regions face pronounced seasonal fluctuations in temperature, food availability, and biotic interactions. To survive, insects must synchronize their life cycles with essential resources and endure unfavorable conditions. Diapause, a programmed developmental arrest coupled with metabolic suppression, is a key adaptation that enables insects to overwinter and align their life histories with environmental cycles.

The domestic silkworm, *Bombyx mori*, is both an economically vital species and a Lepidopteran model organism, exhibiting egg diapause as its dormancy strategy. The regulation of silkworm diapause involves a complex endocrine mechanism. The landmark discovery of its endocrine control occurred in the early 1950s, when Fukuda and Hasegawa independently identified the suboesophageal ganglion (SG) as the source of a hormonal regulator [1,2]. Fukuda designated its secretory product as the “diapause factor”, while Hasegawa termed it “diapause hormone” (DH), with the latter becoming the standard terminology. Subsequent biochemical characterization revealed DH as a 24-amino acid neuropeptide with a crucial C-terminal amidation [3,4], whose biological activity essentially depends on its conserved FXPRL-NH_2_ motif [3]. Modern transcriptomic approaches have further demonstrated that synthetic DH administration in non-diapause silkworm pupae reprograms diapause-associated gene expression profiles, confirming that DH orchestrates embryonic diapause initiation through comprehensive regulation of downstream gene networks [5].

Insect diapause involves extensive metabolic reprogramming, with particularly pronounced modifications in lipid and carbohydrate metabolism that promote environmental adaptation [6,7]. Diapause-destined individuals consistently accumulate higher lipid reserves than their non-diapausing counterparts. This pattern has been documented across diverse taxa, including yellow fever mosquito (*Aedes aegypti*) eggs [8], five-spot burnet (*Zygaena trifolii*) larvae [9], flesh fly (*Sarcophaga crassipalpis*) pupae [10], and northern house mosquito (*Culex pipiens*) adults [11]. During diapause, insects also undergo distinctive carbohydrate metabolic reprogramming to enhance environmental adaptation, including the conversion of glycogen into cryoprotective polyols (e.g., sorbitol and glycerol) via the polyol pathway [12]. Sorbitol enhances freeze tolerance, regulates osmotic balance, and protects embryos under low-temperature stress. This process is primarily catalyzed by sorbitol dehydrogenase (SDH), a key enzyme in metabolic homeostasis. Upon diapause termination, often triggered by chilling at 5°C, SDH mediates the NAD-dependent reconversion of sorbitol back into glycogen [13]. In *B. mori*, three *Sdh* genes have been identified: *BmSdh1*, *BmSdh2a*, and *BmSdh2b*. Among these, *BmSdh2a* and *BmSdh2b* demonstrate striking sequence similarity, with nearly identical nucleotide sequences and highly conserved amino acid sequences [14]. Due to this exceptional structural and functional conservation, they are generally regarded as allelic variants of the same genetic locus and are conventionally referred to collectively as *BmSdh2* in studies of sorbitol metabolism. SDH activity and *BmSdh2* expression increase significantly when diapause embryos are exposed to low temperatures [14]. Notably, *BmSdh2* expression is markedly altered in both diapause and non-diapause strains following exposure to diapause-inducing factors, including DH injection or DH gene overexpression [15–17], suggesting its critical role in determining post-fertilization diapause status. Although SDH-mediated sorbitol metabolism has been extensively studied from physiological and biochemical perspectives [14,18], its molecular mechanisms and potential crosstalk with lipid metabolic pathways during diapause remain poorly understood. In particular, the role of lipid metabolism and its integration with carbohydrate regulation in diapause induction and maintenance warrants further exploration.

In this study, we successfully induced transgenerational diapause in a non-diapause silkworm strain, observing diapause initiation within 24 h after DH injection. Transcriptome analysis identified *BmSdh2* as a key candidate gene, displaying significant expression changes upon diapause induction. Utilizing CRISPR/Cas9-mediated knockout of *BmSdh2*, we demonstrated that its dosage acts as a critical determinant of the transgenerational diapause phenotype. Our findings reveal a novel and essential role of *BmSdh2* as a molecular switch in the silkworm diapause regulatory pathway, while also providing new insights into the metabolic integration between carbohydrate and lipid metabolism underlying diapause regulation.

## Results

### A peak of inducing a diapause-like state occurs at 24 h post-DH injection

**Fig 1:**
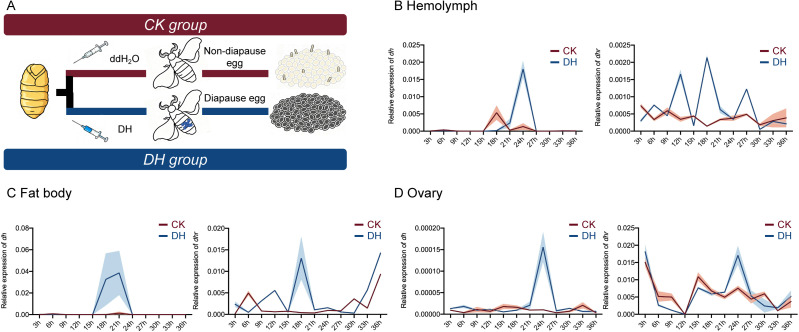
Establishment of a system to study the transmission of diapause hormone in the hemolymph-fat body-ovary of silkworm pupae. (A) Schematic diagram of diapause assay design. The polyvoltine silkworm strain *Nistari*, which naturally lays non-diapause eggs under normal conditions, was used as the control group (CK group). Female pupae were injected with diapause hormone (DH) on the third day of the pupal stage, resulting in the laying of diapause eggs as the treatment group (DH group). (B-D) Temporal expression profiles of *dh* (left) and its receptors *dhr* (right) in the hemolymph (B), fat body (C), and ovaries (D) of the control and treatment groups following DH injection. Three biological replicates were performed. The shaded regions represent means ± SEM.

Previous studies have suggested that the diapause fate in silkworm offspring is determined by oocytes at day 3 of the pupal stage [19,20]. However, the precise timing of diapause induction in silkworms remains unclear. In the non-diapause strain, *Nistari*, DH has been shown to induce embryonic diapause. To explore the dynamics of DH transmission across the hemolymph-fat body-ovary axis during diapause induction, DH (10 µg) was injected into female *Nistari* pupae at 36 h into the pupal stage. The resulting moths laid brown diapause eggs (Fig 1A), confirming successful DH-induced diapause in *Nistari*.

To investigate diapause induction mechanisms in this non-diapause strain, we compared peak reprogramming responses post-DH injection between induced diapause and non-diapause conditions. The mRNA expression levels of the gene encoding DH (*dh*) and its receptor gene (*dhr*) were detected in silkworm hemolymph, fat body, and ovarian tissues after DH injection. In hemolymph, *dh* expression peaked at 24 h post-injection, while *dhr* expression was significantly higher in the treatment group compared to the control group at 12, 18, and 27 h post-injection (Fig 1B). In fat body, *dh* expression peaked at 18 and 21 h post-injection, while *dhr* expression peaked at 18 h post-injection (Fig 1C). At these times, the expression levels of both *dh* and *dhr* in the treatment group were significantly elevated compared to the control group.

To explore the physiological and biochemical effects of DH and DHR binding on oocytes, ovarian tissue samples were collected to measure the relative expression levels of *dh* and *dhr*. In the ovary, the relative expression of *dh* peaked at 24 h post-injection. The expression pattern of *dhr* was similar in both the control and treatment groups. However, at 24 h, the expression level in the control group was significantly higher than that in the treatment group (Fig 1D). Based on the observed trends in *dh* and *dhr* expression in female pupae from 3 to 36 h after DH injection, we speculated that 24 h might represent a critical time point for DH action.

### The expression of *BmSdh2* was up-regulated in diapause embryos induced by DH

**Fig 2:**
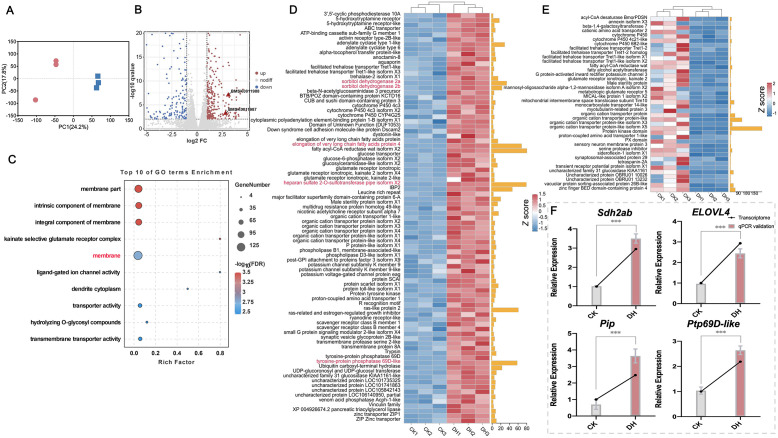
Gene regulation in the maternal ovary during diapause initiation. (A) Principal component analysis (PCA) of log2-transformed transcripts per million (TPM) for all genes. Red dots denote samples from the control group, while blue squares represent samples from the DH treatment group. (B) Volcano plot of significantly differentially expressed genes (DEGs) in the ovarian transcriptome of the DH treatment group compared to the control group (red, up-regulated; blue, down-regulated; gray, no difference). (C) Gene ontology enrichment analysis highlighting the biological processes most strongly associated with DEGs involved in diapause determination regulated by the maternal ovary. (D and E) Heatmaps of the up-regulated (D) and down-regulated (E) DEGs enriched in the membrane pathway across different samples. *Z*-scores are derived from the normalized expression value of each gene (TPM). The yellow bars on the left indicate the (-log10 *q*-value). (F) Fold change of selected diapause-related DEGs quantified by qRT-PCR analysis. Data are presented as means ± SEM (n = 3; *** *P* < 0.001, two-tailed Student’s *t*-test).

In silkworms, embryonic diapause is initiated through specific DHR expression in the ovary triggered by DH [21,22], though the underlying molecular mechanisms remain unclear. To investigate gene regulation during diapause induction, RNA sequencing (RNA-seq) was performed on ovarian tissue collected 24 h post-DH injection in the non-diapause strain *Nistari*. Principal component analysis (PCA) showed a distinct separation in gene expression profiles between control and DH-treatment groups (Fig 2A). A total of 477 differentially expressed genes (DEGs) were identified in the DH-treated group compared to the control group, with 303 up-regulated and 174 down-regulated (Fig 2B and S1 Table). The ovarian tissue of the DH-treated group exhibited a significant increase in up-regulated genes at 24 h post-injection, indicating that the up-regulation of *dh* and its receptor *dhr* in the ovaries activates downstream diapause-related genes. This finding indicates that enhanced expression of these genes plays a critical role in inducing or maintaining the diapause phenotype of offspring.

To investigate the relevance of the DEGs identified above, we performed Gene Ontology (GO) enrichment analysis. The DEGs were categorized into three primary GO domains: biological process, cellular component, and molecular function. The top 10 enriched GO terms are illustrated in Fig 2C. The GO enrichment analysis revealed that diapause-related genes were predominantly associated with membrane-related pathways, including membrane, membrane part, intrinsic component of membrane, and integral component of membrane. Notably, 125 DEGs were implicated in membrane-related processes, prompting further analysis of these genes as potential candidates involved in diapause regulation (Fig 2C and S2 Table).

The up- and down-regulation of DEGs enriched in the membrane pathway were analyzed, and a corresponding heatmap was generated (Fig 2D and 2E). Particular attention was given to genes that exhibited a significant up-regulation in the ovary in response to increased *dh* expression. Based on the expression level and statistical significance, five key genes from the membrane pathway enriched DEGs were selected for further validation: *sorbitol dehydrogenase 2a* (*Sdh2a*), *sorbitol dehydrogenase 2b* (*Sdh2b*), *elongation of very long chain fatty acids protein 4* (*ELOVL4*), *heparan sulfate 2-O-sulfotransferase pipe* isoform X2 (*Pip*), and *tyrosine-protein phosphatase 69D*-*like* (*Ptp69D-like*) (Fig 2D and 2F). Notably, the nucleotide sequences of *Sdh2a* (BMSK0011987) and *Sdh2b* (BMSK0011988) share a high degree of similarity [14], which posed challenges in designing specific primers for individual verification. Consequently, the common region of these genes was validated collectively as *Sdh2ab*. The qRT-PCR results revealed significant differences in the expression of the five DEGs, which aligned closely with the RNA-seq expression patterns, confirming the authenticity and reliability of the transcriptome data and supporting their use for subsequent analysis (Fig 2F). Based on gene function annotation, the *Sdh2ab* gene emerged as a promising candidate for identifying the embryonic diapause gene.

### Phylogenetic analysis and gene characterization of *BmSdh2*

**Fig 3:**
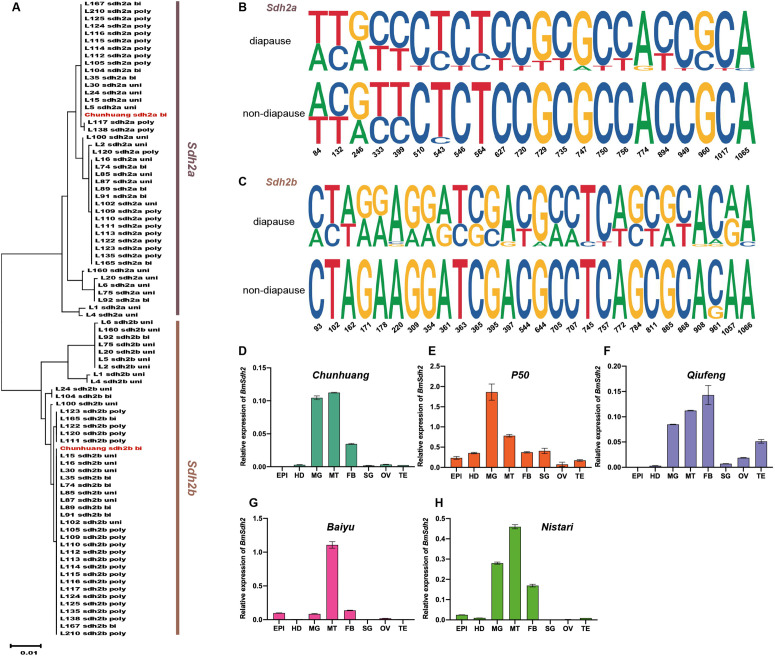
Phylogenetic analysis, sequence logos, and expression patterns of *BmSdh2* in diapause and non-diapause strains. (A) Phylogenetic analysis of nucleotide sequences of *BmSdh2a* and *BmSdh2b* genes across different silkworm strains. The maximum-likelihood tree was generated using the T92 + G + I model selected by the Akaike information criterion, with 1,000 bootstrap replicates. Except for the *Chunhuang* strain identified in this study, each sample is represented by sample IDs. Detailed sample information can be found in S3 Table. Abbreviations: uni, univoltine strain (diapause); bi, bivoltine strain (diapause); poly, polyvoltine (non-diapause). (B and C) Sequence logos of *BmSdh2a* (B) and *BmSdh2b* (C) genes from diapause and non-diapause strains. (D-H) Spatial expression patterns of *BmSdh2* mRNA levels in eight tissues on the third day of the fifth-instar larvae stage. *BmRp49* was used as an internal reference. Abbreviations: EPI, epidermis; HD, head; MG, midgut; MT, Malpighian tubule; FB, fat body; SG, silk gland; OV, ovary; TE, testis. Three biological replicates were performed, and data are presented as means ± SEM.

To investigate the evolutionary relationships of *BmSdh2* in silkworms, we performed phylogenetic analysis using nucleotide sequences from 43 silkworm strains (16 univoltine, 9 bivoltine, and 18 polyvoltine strains; S3 Table). The phylogenetic tree revealed two distinct branches corresponding to *BmSdh2a* and *BmSdh2b* (Fig 3A). The sequences from the diapause bivoltine strain *Chunhuang* properly clustered within their respective branches, confirming the identity of our sequenced fragments as *BmSdh2*. However, no apparent correlation was observed between voltinism and clustering of branches in the internal gene analysis. This lack of correlation might be attributed to the minimal sequence variation among strains. To investigate further, we analyzed single nucleotide polymorphism (SNP) sites between diapause and non-diapause strains and visualized the results using Logomaker [23]. The sequence logos of *BmSdh2a* (Fig 3B) and *BmSdh2b* (Fig 3C) revealed striking patterns: in *BmSdh2a*, diapause strains (univoltine and bivoltine) consistently showed a “TTGCC” motif in the first five positions, while non-diapause strains (polyvoltine) exhibited an “ACGTT” pattern. For *BmSdh2b*, diapause strains showed extensive nucleotide polymorphisms, whereas non-diapause strains showed only a single base transversion from C to G at position 961. These findings indicated that *BmSdh2* in diapause strains possesses a significantly higher abundance of SNPs than in non-diapause strains, accompanied by distinct expression patterns.

To further analyze *BmSdh2* expression patterns in wild-type (WT) silkworms, qRT-PCR analysis was performed. Spatial expression analysis was performed using diapause strains (*Chunhuang*, *P50*, *Qiufeng*, *Baiyu*) and the non-diapause strain *Nistari.* The mRNA expression levels of *BmSdh2* were examined in 8 different tissues, including epidermis, head, midgut, Malpighian tubules, fat body, silk gland, ovary, and testis on day 3 of the fifth larval instar (L5D3). The results revealed that *BmSdh2* was highly expressed in the midgut, Malpighian tubules, and fat body across the tested strains compared to other tissues (Fig 3D-3H). Notably, in the *Baiyu* strain, *Sdh2* expression was significantly higher in the Malpighian tubules than in the midgut and fat body, a pattern distinct from the other strains (Fig 3G). We speculated that this unique expression profile may be attributed to *Baiyu* being the only Japanese strain among the detected strains.

### *BmSdh2*^*+*/*−*^ and *BmSdh2*^*−*/*−*^ mutants were generated in diapause silkworm using CRISPR-Cas9

**Fig 4:**
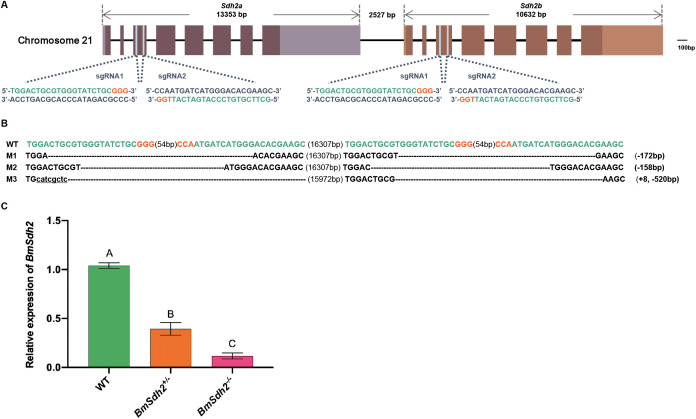
Construction of loss-of-function mutants of *BmSdh2* using the CRISPR/Cas9 system. (A) Schematic diagram of *BmSdh2* gene structure and sgRNA target sites. Dark boxes indicate coding exons, while light boxes indicate untranslated regions (UTR). The two target sites are both located on exon 3 of the *BmSdh2a* and *BmSdh2b* genes. The sequences targeted by the sgRNAs are highlighted in green, and the protospacer adjacent motif (PAM) sequences are shown in red. (B) Homozygous mutations induced by the CRISPR/Cas9 system. The WT sequence is displayed at the top. The PAM sequence is marked in red; dotted lines indicate deleted residues; underlined lowercase letters represent inserted residues; and the number of altered nucleotides is indicated on the right. (C) mRNA expression levels of *BmSdh2* in WT, *BmSdh2*^*+/−*^, and *BmSdh2*^*−/−*^ mutants at the larval stage. Three biological replicates were performed. Error bars represent means ± SEM; bars labelled with different letters indicate significant differences between samples (*P* < 0.01). *P*-values for pairwise comparison were derived from two-tailed Student’s *t*-test.

To investigate the role of *BmSdh2* in diapause induction, we generated knockout mutants using CRISPR-Cas9 mediated genome editing in the diapause (bivoltine) strain *Chunhuang*. The *BmSdh2* gene is located on chromosome 21 and consists of eight exons. Given the high sequence similarity between *BmSdh2a* (BMSK0011987) and *BmSdh2b* (BMSK0011988), we designed two target sites, both located in exon 3 (Fig 4A). This approach enabled us to generate double knockout mutants (*BmSdh2*^*+/−*^) for both *BmSdh2a* and *BmSdh2b.* Subsequently, homozygous mutants (*BmSdh2*^*−/−*^) were obtained through multiple rounds of self-crossing.

Sequencing analysis revealed a range of mutational alterations in *△BmSdh2* mutants (Fig 4B). In parallel, qRT-PCR analysis demonstrated that the relative transcript levels of *BmSdh2* in heterozygote were significantly lower compared to the WT, while transcription was completely down-regulated in homozygous mutants (Fig 4C). These findings collectively confirm the successful generation of *BmSdh2* mutants in the silkworm.

### The role of *BmSdh2* in silkworm embryonic diapause is dosage-dependent

**Fig 5:**
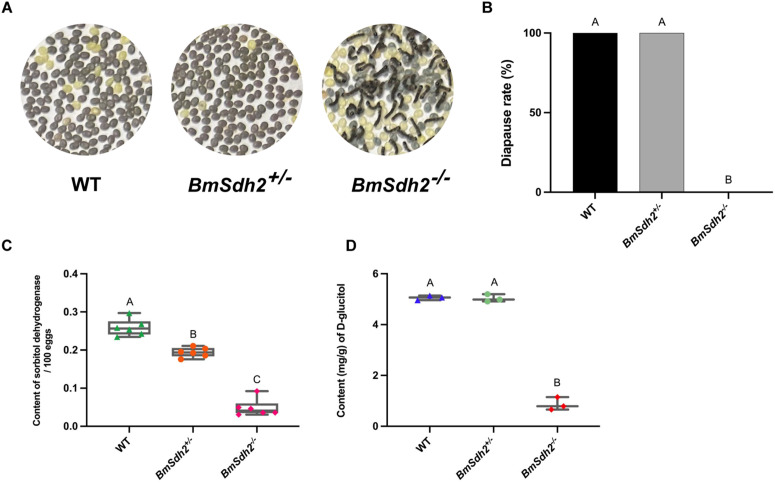
Dosage-dependent regulation of embryonic diapause by *BmSdh2* in *B. mori.* (A) Embryonic diapause phenotype of CRISPR/Cas9-mediated *BmSdh2* knockout mutants. Homozygous *BmSdh2* knockout lines produced non-diapause eggs at 25°C, while the WT bivoltine strain (*Chunhuang*) and heterozygous *BmSdh2* knockout lines generated diapause eggs when maternal embryos were incubated at 25°C. (B) Diapause rates of offspring eggs from WT, *BmSdh2*^*+/−*^, and *BmSdh2*^*−/−*^ mutants at an incubation temperature of 25°C. (C) Sorbitol dehydrogenase content per 100 eggs in WT, *BmSdh2*^*+/−*^, and *BmSdh2*^*−/−*^ mutants. (D) Quantification of D-glucitol content in offspring eggs of *BmSdh2*^*+/−*^ and *BmSdh2*^*−/−*^ mutants by LC-MS/MS analysis. Six biological replicates were performed. Bars labelled with different letters indicate significant differences between samples (*P* < 0.01). WT, *BmSdh2*^*+/−*^, and *BmSdh2*^*−/−*^ represent wild-type, heterozygous mutants, and homozygous mutants, respectively.

We revealed that WT moths produced 100% diapause embryos, whereas homozygous mutant silkworms *BmSdh2*^*−/−*^ laid 100% non-diapause embryos. In contrast, heterozygous mutant silkworms *BmSdh2*^*+/−*^ showed no significant impact on embryonic diapause occurrence (Fig 5A and 5B). Notably, a marked phenotypic difference was observed between heterozygote and homozygote mutants.

To better understand the difference in diapause incidence between *BmSdh2*^*+/−*^ and *BmSdh2*^*−/−*^ mutants, we assessed the SDH level and its related metabolite, D-glucitol, using enzyme-linked immunosorbent assay (ELISA) and LC-MS/MS, respectively. ELISA results showed that eggs from heterozygous mutants had substantially lower SDH levels compared to WT, with even more pronounced reductions in homozygous mutants (Fig 5C). Concurrently, LC-MS/MS analysis demonstrated that non-diapause embryos from *BmSdh2*^*−/−*^ silkworms exhibited markedly lower D-glucitol levels than diapause embryos from *BmSdh2*^*+/−*^ mutants (Fig 5D). In contrast, the D-glucitol levels in *BmSdh2*^*+/−*^ mutants were slightly lower than those in WT silkworms. The differences in D-glucitol content between *BmSdh2*^*+/−*^ and *BmSdh2*^*−/−*^ mutants correlated with variations in diapause incidence in silkworm embryos. This suggests that a single copy of *BmSdh2* is sufficient for its normal function, a phenomenon consistent with a gene dosage effect, whereas the complete loss of the gene results in a loss of function.

### Diapause was accompanied by lipid content enrichment

**Fig 6:**
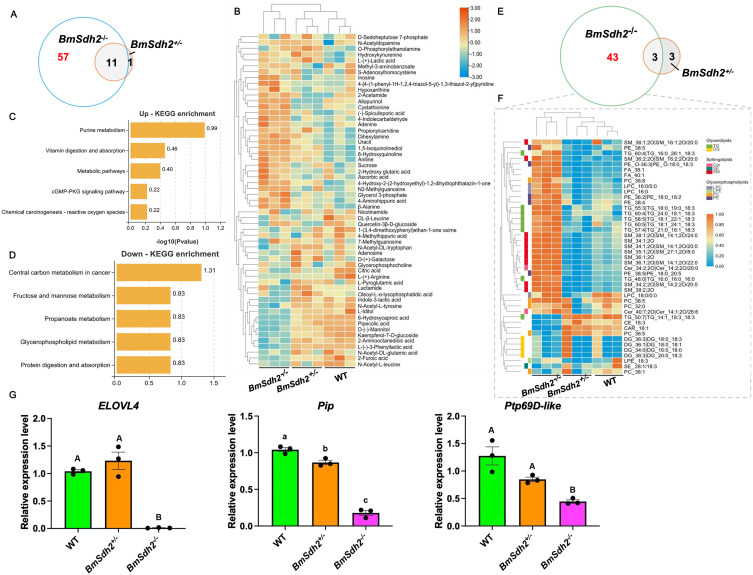
Metabolic atlas of silkworm embryonic diapause revealed by metabolomics and lipidomics. (A) Venn diagram showing number of differentially accumulated metabolites (DAMs) commonly and specifically regulated by *BmSdh2*^*−/−*^ (left, blue) and *BmSdh2*^*+/−*^ (right, purple) compared to WT. (B) Hierarchical clustering of selected diapause-related DAMs across different samples. (C and D) KEGG enrichment analysis of up-regulated (C) and down-regulated (D) DAMs associated with silkworm diapause. (E) Venn diagram illustrating number of differentially abundant lipids (DALs) commonly and specifically regulated by *BmSdh2*^*−/−*^ (left, green) and *BmSdh2*^*+/−*^ (right, purple) relative to WT. (F) Hierarchical clustering of selected diapause-related DALs in different samples. (G) Analysis of relative expression levels of key diapause-associated lipid metabolism genes (*ELOVL4*, *Pip*, and *Ptp69D-like*). Data are presented as mean ± SEM (n = 3). Different uppercase letters (A, B, C) indicate significant differences between groups at *P* < 0.01, while different lowercase letters (a, b, c) indicate significant differences at *P* < 0.05. WT, *BmSdh2*^*+/−*^, and *BmSdh2*^*−/−*^ represent wild-type, heterozygous mutants, and homozygous mutants, respectively.

To investigate the metabolite changes in embryos caused by *BmSdh* mutation, we integrated metabolomics and lipidomics to construct a comprehensive metabolic atlas of the silkworm embryo diapause process. In metabolomics analysis, the *BmSdh2*^*−/−*^ group showed 68 differentially accumulated metabolites (DAMs), while the *BmSdh2*^*+/−*^ group showed only 12 compared to the WT group. Among them, 11 DAMs were common to both *BmSdh2*^*−/−*^ and *BmSdh2*^*+/−*^ mutants (Fig 6A). The 57 unique DAMs in the *BmSdh2*^*−/−*^ mutants were used to generate a heatmap, revealing significant metabolite alterations in non-diapause eggs (Fig 6B). We performed KEGG analysis on DAMs. The up-regulated metabolites were primarily enriched in pathways such as purine metabolism, vitamin digestion and absorption, and general metabolic pathways, while the down-regulated metabolites were mainly associated with central carbon metabolism in cancer (Fig 6C and 6D). These findings suggest that non-diapause eggs exhibit enhanced activation of energy supply and metabolic regulation.

To investigate the impact of the diapause state on lipid metabolism, we compared the lipidomic profiles of *BmSdh2*^*−/−*^, *BmSdh2*^*+/−*^ and WT samples using the OPLS-DA method. The analysis revealed clear separation between the *BmSdh2*^*−/−*^ mutants and the WT group, as well as between the *BmSdh2*^*+/-*^ mutants and the WT group (S1 Fig). Specifically, 43 unique lipid species were identified as differentially abundant lipids (DALs), which showed significant alterations in the *BmSdh2*^*−/−*^ mutants compared to the *BmSdh2*^*+/−*^ mutants and the WT group (Fig 6E and 6F). Among these DALs, 11 were down-regulated, while the remaining 32 lipid species were up-regulated in the offspring eggs of *BmSdh2*^*−/−*^. In contrast, the *BmSdh2*^*+/−*^ samples showed no significant effect on the lipid profiles in the offspring embryos compared to the WT group. We further examined the expression of key lipid metabolism-related genes (*ELOVL4*, *Pip*, and *Ptp69D-like*) that were initially identified alongside *BmSdh2* through transcriptomic screening. Notably, these genes all play roles in lipid metabolic processes and exhibited significantly down-regulated expression in non-diapause eggs compared to diapause eggs (Fig 6G), reinforcing the close association between lipid metabolism and diapause termination. The findings align with previous studies that have indicated that the termination of diapause is accompanied by lipid content enrichment, a process essential for insect development and adaptation to stressful environments [24].

## Discussion

**Fig 7:**
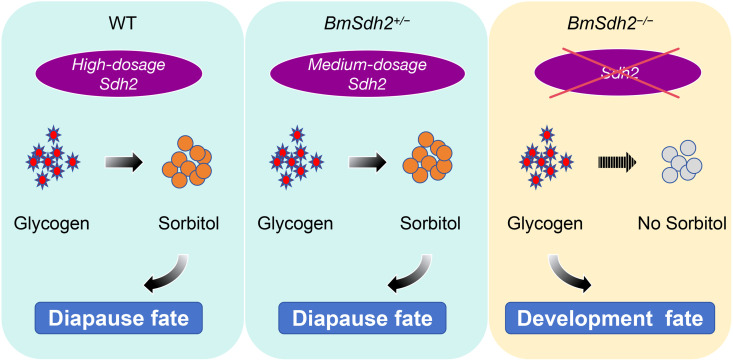
Proposed model of dosage-dependent diapause regulation by *BmSdh2* in the silkworm. In WT with high *BmSdh2* expression, glycogen is converted to high levels of sorbitol, enabling entry into diapause fate; in heterozygous mutants (*BmSdh2*^*+/−*^) with medium *BmSdh2* dosage, sufficient sorbitol accumulation still supports completion of diapause; In contrast, homozygous mutants (*BmSdh2*^*−/−*^) lacking functional SDH exhibit a protective metabolic bypass: recognizing the inability to convert accumulated sorbitol to fructose (which would irreversibly trap embryos in diapause), the system prevents sorbitol accumulation altogether, resulting in direct progression to developmental fate without undergoing diapause. This dosage-dependent regulation demonstrates how SDH activity gates entry into the diapause program through sorbitol metabolic sensing.

Our study demonstrates that BmSDH regulates silkworm embryonic diapause in a dose-dependent manner. Complete knockout (*BmSdh2*^*−/−*^) entirely terminated diapause, whereas partial knockout (*BmSdh2*^*+/−*^) had no effect, suggesting that a single functional *BmSdh2* copy (haplo-sufficiency) is sufficient for normal diapause. Biochemical analysis confirmed significantly reduced D-glucitol levels in *BmSdh2*^*−/−*^ embryos, indicating that full knockout disrupts diapause through an epistatic effect, further supporting dosage dependence (Fig 7). Additionally, we observed coordinated expression changes between *BmSdh2* and key lipid metabolism genes (*ELOVL4*, *Pip*, *Ptp69D-like*) in diapause embryos (Fig 2F). Further supporting this connection, our qRT-PCR analysis revealed that all three lipid metabolism genes were significantly down-regulated in non-diapause eggs compared to diapause eggs (Fig 6G). Among these, *ELOVL4* may help compensate for SDH deficiency by maintaining cell membrane flexibility through its role in very-long-chain fatty acid synthesis.

In previous studies, the expression level of *BmSdh2* was exclusively examined during the embryonic stage [14]. Here, we investigated the spatial expression patterns of *BmSdh2* during the larval stage across different silkworm strains. Regrettably, no tissue-specific expression differences were identified between diapause and non-diapause strains, potentially due to limited non-diapause strain materials, with only *Nistari* included. Through the phylogenetic analysis, we found that *BmSdh2* gene in diapause strains exhibits significantly higher SNP abundance compared to non-diapause strains, along with distinct expression patterns. This observation is consistent with the evolutionary trajectory of silkworm strains, which transitioned from diapause type (univoltine, bivoltine) to non-diapause type (polyvoltine) [25]. Future studies combining targeted lipidomics with genetic perturbations of *ELOVL4*, *Pip*, and *Ptp69D-like*, all of which were significantly down-regulated in non-diapause eggs (Fig 6G), will help disentangle these interactions and clarify how lipid metabolism networks coordinate with SDH-mediated sugar-alcohol homeostasis to regulate diapause.

Sorbitol, a key compound of the polyol pathway, plays a critical role in maintaining glucose-fructose balance [26]. During diapause formation, antifreeze substances, such as sorbitol and glycerol accumulate in silkworm eggs to facilitate low-temperature survival [12]. During diapause termination, SDH converts sorbitol to fructose in embryonic cells, which is further transformed into glucose, leading to glycogen accumulation. This process supports egg cell differentiation and growth [14,27]. Research in *Drosophila* has shown that deletion of the SDH-encoding gene *SORD* leads to severe neurological defects, including synaptic degeneration and progressive motor dysfunction [28,29]. In contrast, our silkworm model revealed a different yet equally striking phenotype: *BmSdh2*^*−/−*^ mutants exhibited transgenerational alterations in diapause characteristics, while *BmSdh2*^*+/−*^ heterozygotes retained normal diapause phenotypes. This dosage-dependent effect aligns with our proposed model (Fig 7), wherein wild-type silkworms employ SDH to accumulate sorbitol during diapause initiation, followed by SDH-mediated sorbitol to glycogen conversion after prolonged cold exposure. Complete SDH loss in knockout mutants appears to trigger a protective mechanism that blocks sorbitol accumulation, thereby impairing diapause induction. Crucially, the phenotypic similarity between heterozygous and wild-type individuals (Fig 5A) supports a permissive threshold of SDH activity for proper diapause regulation. This threshold model not only explains the stable diapause phenotype observed in specialized strains but also accounts for the abrupt phenotypic disruption seen exclusively in complete knockout. The dosage-sensitive nature of SDH function in diapause regulation thus operates through a sophisticated mechanism that ensures phenotypic stability across a range of enzyme activities while eliciting drastic effects only under extreme deficiency. Furthermore, the dosage dependence of SDH in diapause regulation clarifies why diapause silkworm strains occasionally produce non-diapause eggs, a phenomenon likely tied to insufficient SDH-mediated metabolic control.

In summary, our study presents the first molecular functional analysis of *BmSdh2*, the silkworm ortholog of *Drosophila SORD*. We identified *BmSdh2* as a diapause-related gene in silkworms and demonstrated that its dosage can modify the diapause phenotype of offspring. These findings establish a foundation for exploring DH signaling pathways in silkworm diapause and hold significant economic potential for the sericulture industry. Gene editing approaches to regulate silkworm diapause offer a promising strategy, combining cost-effectiveness with operational convenience.

## Materials and methods

### Silkworm strains

In this study, we utilized both non-diapausing polyvoltine (*Nistari*) and diapausing bivoltine (*P50*, *Chunhuang*, *Qiufeng*, and *Baiyu*) silkworm strains. The rearing condition for the insects was as previously described [30]. Specifically, silkworm larvae were fed fresh mulberry leaves and maintained under standard conditions at 25˚C with a 12-h light/dark cycle and relative humidity of 65%-75%.

### Injection of diapause hormone

The silkworm DH peptide is a neuropeptide consisting of 24 amino acids, with the following sequence: Thr-Asp-Met-Lys-Asp-Glu-Ser-Asp-Arg-Gly-Ala-His-Ser-Glu-Arg-Gly-Ala-Leu-Cys-Phe-GIy-Pro-Arg-Leu-NH_2_ [3]. The DH used in this experiment (sequence: TDMKDESDRGAHSERGALCFGPRL-NH_2_) was synthesized by Sangon Biotech Co., Ltd. (Shanghai, China). Injections were administered to female pupae at the intersegment membrane of the third abdominal segment 36 h after pupation. Previous studies have demonstrated that a diapause rate of approximately 100% is achieved when pupae are supplemented with 10 μg of DH [5]. Therefore, to induce optimal diapause effects, pupae of the non-diapausing strain *Nistari* in the treatment group were injected with 10 μg of DH, whereas those in the control group were injected with UltraPure DNase/RNase-Free water. The silkworm pupae were maintained under the same rearing conditions as the larvae, after which they were allowed to mate and lay eggs as adults. The offspring embryos of the control group were incubated at 25°C for 10 d until larvae hatched, whereas those of the treatment group enter a diapause state due to the DH injection. Hemolymph, fat body, and ovarian tissue were collected into phosphate-buffered saline at 3-h intervals from 3 to 36 h post-injection. For each time point, tissues from three individual silkworms were pooled into a single sample for analysis.

### RNA isolation, cDNA synthesis and quantitative real-time PCR (qRT-PCR)

Total RNA was isolated from both the control group and the DH injection treatment group using the TRIeasy Total RNA Extraction Reagent (Yeasen, China) in accordance with the manufacturer’s protocol. RNA concentration was quantified by spectrophotometer absorbance, and integrity was confirmed by gel electrophoresis analysis. Subsequently, 1 μg of total RNA was used to synthesize cDNA using PrimeScript RT reagent Kit with gDNA eraser (Takara, Dalian, China). Quantitative real-time RT-PCR (qRT-PCR) was conducted on the Step OnePlus Real-Time PCR system (Applied Biosystems) with a SYBR Green Real-Time PCR master mix (Yeasen, China). To establish standard curves, a 5-fold serial dilution of pooled cDNA was utilized as the template. Relative quantification of gene expression was performed using the 2^-∆∆Ct^ method [31], with the ribosomal protein 49 gene (*rp49*) serving as an internal control for normalization. The thermal cycling protocol included an initial denaturation step at 95°C for 5 min, followed by 40 cycles of denaturation at 95°C for 10 s and annealing/extension at 60°C for 30 s. Sequences of all qRT-PCR primers are provided in S4 Table.

### RNA sequencing (RNA-seq) analysis

Total RNA was isolated from the ovaries of individual animals 24 h post-injection of either diapause hormone or ddH_2_O. Three biological replicates were sequenced for both the control and treatment groups. For mRNA sequencing, the total RNA was initially enriched and subsequently fragmented to prepare for cDNA synthesis and library construction. The constructed library was sequenced on the Illumina 2000 platform. Raw sequencing data were processed for quality control, filtered, alignment, and quantification using FastQC, Trimmomatic, Bowtie2, and Rsem, respectively, with reference to the *B. mori* genome database (available at https://silkdb.bioinfotoolkits.net) [32–35]. mRNA abundance was normalized using Deseq2 [36]. Gene expression levels were calculated and employed to identify differential gene expression between samples (Y/X). Differentially expressed genes (DEGs) were identified using the Poisson Distribution Method, with a false discovery rate (FDR) < 0.05 and an absolute log_2_(Y/X) value > 1. Function annotations and enrichment analysis of DEGs were performed using the DAVID tool [37], with gene ontology (GO) terms. Visualization of the bioinformatic analysis was carried out using the OmicShare tools available at www.omicshare.com/tools.

### Phylogenetic and sequence alignment analysis

Phylogenetic and sequence alignment analysis were performed using MEGA 11 [38]. A phylogenetic tree of *Sdh2* sequences from silkworms was reconstructed using the Maximum likelihood (ML) method under the T92 + G + I model, which was selected based on the Akaike information criterion. The bootstrap test (1,000 replicates) was performed to determine the percentages of replicate trees in which associated taxa clustered together, as described previously [39]. Evolutionary distances were calculated using the Poisson correction method and are expressed as the number of nucleotide substitutions per site. The *Sdh2* sequence from the diapause bivoltine strain *Chunhuang* was obtained in this study, while sequences from other silkworm strains were retrieved from the CNGB Nucleotide Sequence Archive (CNSA) of China National GeneBank DataBase (CNGBdb, https://db.cngb.org) [25,40,41]. Detailed information on these sequences is provided in S3 Table. For sequence alignment analysis, single nucleotide polymorphism (SNP) sites among different diapause-type strains were identified and analyzed using MEGA. Sequence logos were generated using Logomaker [23].

### CRISPR/Cas9-mediated construction of mutants

The CRISPR/Cas9 system was employed to generate the *ΔBmSdh2* mutant. Two 23-nucleotide single-guide RNAs (sgRNA) were designed to target specific exonic regions of *BmSdh2*, adhering to the 5’-GG-N_18_-NGG-3’ rule. Potential CRISPR/Cas9 target sequences were identified by analyzing the full-length cDNA sequence using the CRISPRdirect online tool (https://crispr.dbcls.jp/) [42]. The selected sgRNAs, *BmSdh2*-sgRNA1-TGGACTGCGTGGGTATCTGCGGG (5’-3’) and *BmSdh2*-sgRNA2-GCTTCGTGTCCCATGATCATTGG (5’-3’) were synthesized *in vitro* using the MEGAscript T7 kit (Ambion) following the manufacturer’s protocol. The PTD1-T7-Cas9 plasmid (ViewSolid Biotech) was linearized with the NotI restriction enzyme (Fermentas), and Cas9 messenger RNAs (mRNA) was subsequently synthesized using the mMESSAGE mMACHINE T7 Kit (Ambion) according to the provided instructions. The purified Cas9 mRNA and sgRNAs were stored at -80°C for further use.

Silkworm embryos were collected within 2 h post-oviposition. Following electrical stimulation to break diapause, a mixture containing Cas9 mRNA (300 ng/μL), *BmSdh2*-sgRNA1 (200 ng/μL), and *BmSdh2*-sgRNA2 (200 ng/μL) was microinjected into the embryos. All microinjection procedures were completed within 6 h after oviposition. The injected embryos were then incubated at 25°C in a humidified chamber for approximately 10 d until larvae hatched.

The two specific sgRNAs and Cas9 mRNA were co-injected into early embryos of *B. mori*. To verify the presence of mutations, randomly selected representative F_1_ offspring were subjected to PCR-based analysis and sequencing using gene-specific primers, which confirmed the successful introduction of mutations in these individuals. F_1_ moths carrying chromosomal deletions were then mass-crossed to propagate the mutant allele to the F_2_ generation. From this generation, homozygous mutant individuals were identified and selected to establish the next generation (F_2_), designated as *BmSdh2*^*−/−*^.

### Mutagenesis analysis

Genomic DNA was extracted from *△BmSdh2* and WT silkworm larvae with SDS-mediated lysis followed by phenol extraction. Aliquots containing 200 ng of total DNA were utilized as templates for gene-specific PCR amplification. The resulting amplicons were cloned into the pJET-1.2 vector (Takara, Dalian, China), and randomly selected clones were subjected to Sanger sequencing using gene-specific primers designed to flank the sgRNA target sites. The primer sequences are listed in S4 Table. Additionally, the SDH content in WT, *BmSdh2*^*+/−*^, and *BmSdh2*^*−/−*^ silkworm eggs was quantified using the Insect Sorbitol Dehydrogenase Elisa Detection Kit (Jiangsu Enzyme Label Biotechnology Co., Ltd.).

### LC-MS/MS analysis

Eggs from *BmSdh2*^*−/−*^, *BmSdh2^+/−^*, and WT silkworms were collected and stored at -80°C for subsequent analysis. For each sample, approximately 50 ± 0.5 mg of silkworm eggs was weighed and placed in a homogenizer tube. After grinding, 1.4 mL of methanol and 50 μL of inositol (used as the internal standard) were added. The mixture was vortexed for 15 min at 70°C and then centrifuged at 14,000 × g for 3 min. The supernatant was transferred to a new tube and combined with 2.4 mL of a chloroform/ddH_2_O mixture (1:1.4, v/v). The solution was vortexed again and centrifuged at 6,000 rpm for 15 min. A 500 μL aliquot of the upper polar phase was collected and dried using a centrifugal dryer at 45°C. The concentrated phase was then derivatized by adding 50 μL of BSTFA+TMCS and 100 μL of pyridine, following incubation at 70°C for 30 min. Finally, 100 μL of the supernatant was collected for analysis using LC-MS/MS. The analysis was conducted on an HPLC system coupled to a Triple Quad 4500 mass spectrometer equipped with a Turbo V ion source (both from AB Sciex, USA).

### Metabolomics and lipidomics analysis

A silkworm egg sample weighing 100 ± 0.5 mg was obtained and placed in a homogenizer tube, followed by the addition of 800 μL of ddH_2_O. The samples were homogenized and then kept on ice for 10 min. Subsequently, they were mixed with 3 mL of a prechilled methanol/chloroform mixture (2:1, v/v), vortexed for 30 s, and left at room temperature for 1 h. After adding 1 mL of chloroform and 1 mL of ddH_2_O, the mixture was vortexed again and centrifuged for 10 min at room temperature. The top layer, containing metabolites, was transferred to a new tube and freeze-dried, while the bottom layer, containing lipids, was collected and dried using a termovap sample concentrator. The metabolic extracts were dissolved in an acetonitrile/ddH_2_O solution (1:1, v/v) and the lipid extracts were dissolved in a chloroform/isopropanol/acetonitrile/ddH_2_O solution (20:65:35:5, v/v/v/v). These steps prepared the samples for metabolomic and lipidomic profiling, respectively. Each treatment was performed in triplicate.

Untargeted metabolomic and lipidomic analyses were carried out using an Ultimate 3000 ultra-high-performance liquid chromatograph coupled to a Q Exactive Quadrupole-Orbitrap high-resolution mass spectrometer (Thermo Fisher Scientific). The raw metabolomics data were imported into the Compound Discoverer software (Thermo Fisher Scientific) for processing, while the lipidomics raw data were analyzed using MS-DIAL software [43] for further evaluation.

### Statistical analysis of data

All experiments were conducted with a minimum of three independent replicates. Differences between groups were assessed using the two-tailed Student’s *t*-test. Data are presented as means ± standard errors of the means (SEMs). Statistical significance was denoted by lowercase and uppercase letters in the figures: shared letters indicate no significant difference, while different letters represent significant differences (lowercase: *P* < 0.05; uppercase: *P* < 0.01). All analyses and graphical representations were performed using GraphPad Prism version 10 for Mac (GraphPad Software; graphpad.com).

## Supporting information

S1 Fig Principal component analysis results of lipidomics samples. (A) PCA of the WT and *BmSdh2*^*−/−*^ group samples. D and M represent the WT and *BmSdh2*^*−/−*^, respectively. (B) PCA of the WT and *BmSdh2*^*+/−*^ group samples. D and Z represent the WT and *BmSdh2*^*+/−*^, respectively.
(DOCX)

S1 Table Differentially expressed genes (DEGs) between the control and DH-treatment groups. (XLSX)

S2 Table Differentially expressed genes (DEGs) enriched in the menbrane pathway. (XLSX)

S3 Table Detailed information of silkworm strains used in this study. (XLSX)

S4 Table Primers used in this study. (XLSX)
