# Supplementary material for: Dosage-dependent regulation of embryonic diapause by sorbitol dehydrogenase in the silkworm, Bombyx mori
Source: PLoS Genet. 2025 Oct 30;21(10):e1011933. doi: 10.1371/journal.pgen.1011933 (PMC12594423; doi:10.1371/journal.pgen.1011933)
Supplement: S1 Fig — (A) PCA of the WT and BmSdh2−/− group samples. D and M represent the WT and BmSdh2−/−, respectively. (B) PCA of the WT and BmSdh2+/− group samples. D and Z represent the WT and BmSdh2+/−, respectively. (DOCX) [file pgen.1011933.s001.docx]

**
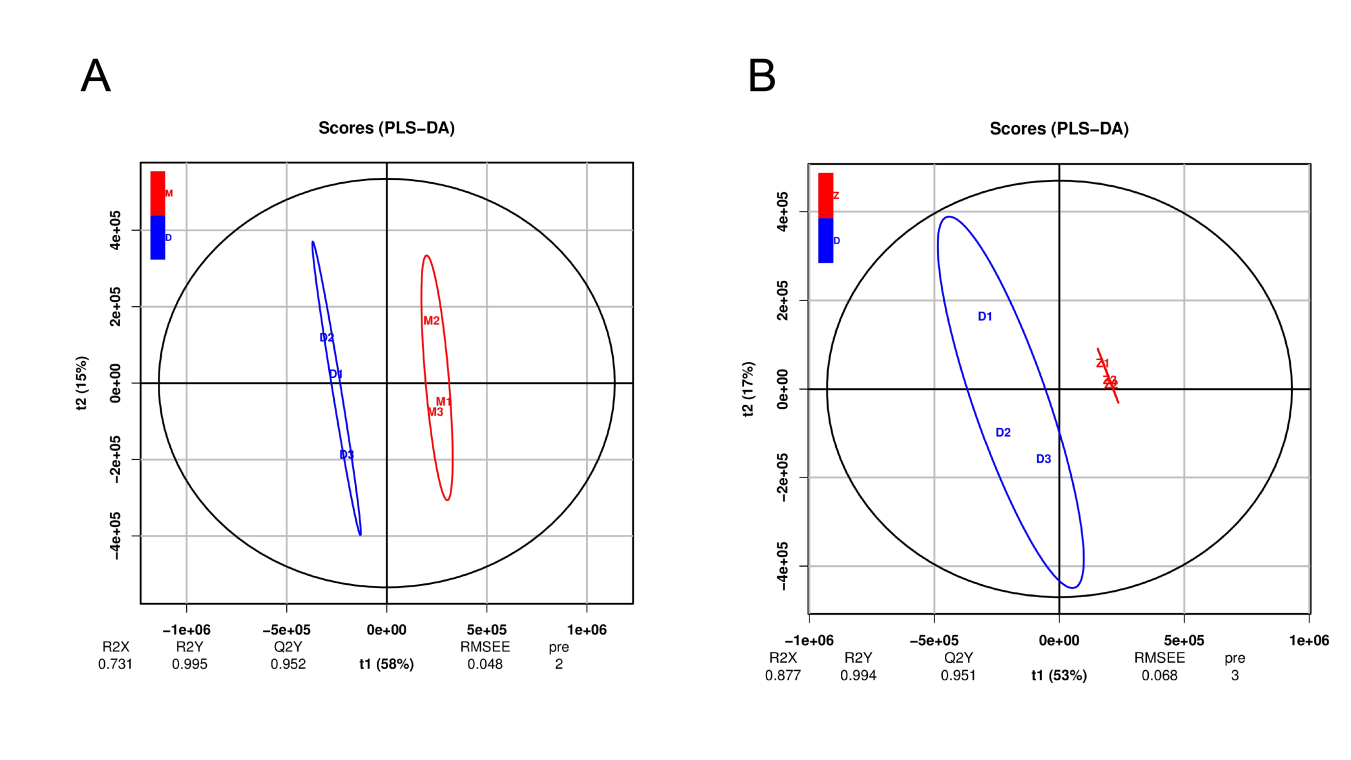
**

**S1 Fig. Principal component analysis (PCA) results of the and lipidomics samples.** (A) PCA of the WT and *BmSdh2^-/-^* group samples. D and M represent the WT and *BmSdh2^-/-^*, respectively. (B) PCA of the WT and *BmSdh2^+/-^* group samples. D and Z represent the WT and *BmSdh2^+/-^*, respectively.
